# Duty factor and foot-strike pattern do not represent similar running pattern at the individual level

**DOI:** 10.1038/s41598-022-17274-0

**Published:** 2022-07-29

**Authors:** Aurélien Patoz, Thibault Lussiana, Bastiaan Breine, Cyrille Gindre, Davide Malatesta

**Affiliations:** 1 Institute of Sport Sciences, University of Lausanne, 1015 Lausanne, Switzerland; 2 Research and Development Department, Volodalen Swiss Sport Lab, 1860 Aigle, Switzerland; 3 Research and Development Department, 39270 Volodalen, Chavéria, France; 4 Research Unit EA3920 Prognostic Markers and Regulatory Factors of Cardiovascular Diseases and Exercise Performance, Health, Innovation Platform, University of Franche-Comté, Besançon, France; 5 Department of Movement and Sports Sciences, Ghent University, 9000 Ghent, Belgium

**Keywords:** Bone quality and biomechanics, Health care

## Abstract

Runners were classified using their duty factor (DF) and using their foot-strike pattern (FSP; rearfoot, midfoot, or forefoot strikers), determined from their foot-strike angle (FSA). High and low DF runners showed different FSPs but DF was assumed to not only reflect what happens at initial contact with the ground (more global than FSP/FSA). Hence, FSP and DF groups should not necessarily be constituted by the same runners. However, the relation between FSP and DF groups has never been investigated, leading to the aim of this study. One hundred runners ran at 9, 11, and 13 km/h. Force data (1000 Hz) and whole-body kinematics (200 Hz) were acquired by an instrumented treadmill and optoelectronic system and were used to classify runners according to their FSA and DF. Weak correlations were obtained between FSA and DF values and a sensitivity of 50% was reported between FSP and DF groups, i.e., only one in two runners was attributed to the DF group supposedly corresponding to the FSP group. Therefore, ‘local’ FSP/FSA and DF do not represent similar running pattern information when investigated at the individual level and DF should be preferred to FSP/FSA when evaluating the global running pattern of a runner.

## Introduction

Runners are usually classified into one of three discrete categories depending on their preferred foot-strike pattern (FSP). A runner is categorized as a (1) rearfoot striker (RFS) when the foot initially contacts the ground with the heel or rear third of the sole, (2) a midfoot striker (MFS) when the heel and toes contact the ground simultaneously, or (3) a forefoot striker (FFS) when the foot initially contacts the ground with the forefoot or front half of the sole^1^. This classification can be obtained using the foot-strike angle (FSA) following the procedure proposed by Altman and Davis^2^. These FSPs involve different neuromuscular activation patterns^3^ and impact attenuation strategies^4–7^. They were also shown to induce different loads on the lower limb and different three-dimensional (3D) stress patterns in the ankle, knee, and hip joints^8–11^, as well as different sagittal plane joint angles during stance^10,12^. Moreover, no differences in running economy have been reported among different FSAs ^13^ or FSPs^14–16^, and changing FSPs is not necessarily recommended for RFS^10,15,17,18^.

More recently, runners have been categorized using the duty factor (DF)^19,20^, i.e., the ratio of ground contact time ($${t}_{c}$$) to stride time [$${t}_{c}$$ + swing time ($${t}_{s})$$], with a higher DF reflecting a greater relative contribution of $${t}_{c}$$ to the running stride^21,22^. Considering both $${t}_{c}$$ and $${t}_{s}$$ simultaneously provides a better understanding of the global running pattern compared with when these temporal variables are considered separately^19,20^. The authors observed that the 20 subjects with highest DF values and 20 subjects with lowest DF values (among a cohort of 54 participants) used different running strategies but had a similar running economy, showing that these two strategies are energetically equivalent at endurance running speeds^19^. A more symmetrical running pattern between braking and propulsion phases in terms of time and vertical center of mass displacement, anterior FSP (MFS and FFS), and extended lower limb during $${t}_{c}$$ at the hip, knee, and ankle joints were observed for low than for high DF runners ^19,20^. On the contrary, high DF runners exhibited greater lower limb flexion during $${t}_{c}$$ at the hip, knee, and ankle joints, more RFS, and less work against gravity to generate forward propulsion than low DF runners^19,20^. Hence, high and low DF runners reflected different FSPs^19,20^, most likely because $${t}_{c}$$ is related to FSP^1,23^. Nonetheless, DF was thought to not only be directly related to the angle at the initial ground contact (via $${t}_{c}$$) as is FSP but to also be functionally representative of a more global running behavior because it takes both the duration of force production ($${t}_{c}$$) and the cycle frequency of running into account^19,20,24^. For this reason, although FSA and DF values should be different among DF (high, mid, and low DF runners) and FSP (RFS, MFS, FFS) groups, respectively, FSP and DF groups should not necessarily be constituted by the same runners. This would confirm that DF should be preferred to FSP/FSA when evaluating the global running pattern of a runner. Nonetheless, to the best of our knowledge, the relationship between the groups created using FSA and DF values has not yet been considered.

Hence, the purpose of the present study was to compare these two different classification methods in analyzing running gait at several running speeds. We hypothesized that (i) FSP groups should have significantly different DF values; (ii) DF groups should have significantly different FSA values; and (iii) FSP and DF groups should not be constituted by the same runners because of weak correlations between FSA and DF values and, thus, leading to weak agreement in the classification of runners between FSP and DF groups.

## Materials and methods

### Participants

One hundred recreational runners, 75 males (age: 31 ± 8 years, height: 180 ± 6 cm, body mass: 70 ± 7 kg, weekly running sessions: 3 ± 2, and weekly running distance: 37 ± 24 km) and 25 females (age: 30 ± 7 years, height: 169 ± 5 cm, body mass: 61 ± 6 kg, weekly running sessions: 3 ± 1, and weekly running distance: 20 ± 14 km), were randomly selected from an existing database consisting of 115 participants^25^ for the purpose of this study. Participants voluntarily participated in the present study, and to be included, they were required to be in good self-reported general health with no current or recent lower-extremity injuries (≤ 1 month), to run at least once a week, and to have an estimated maximal aerobic speed ≥ 14 km/h. The study protocol was approved by the ethics committee of the Vaud canton (commission cantonale d'éthique de la recherche sur l'être humain CER-VD 2020–00334) and adhered to the latest version of the Declaration of Helsinki of the World Medical Association.

### Experimental procedure

After the participants provided written informed consent, retroreflective markers were positioned on the participants (described in Subsec. Data Collection) to record their running biomechanics. For each participant, a 5-s static trial was first recording while he or she stood in a standard anatomical position on an instrumented treadmill (Arsalis T150 – FMT-MED, Louvain-la-Neuve, Belgium) for calibration purposes. Then, a 7-min warm-up run was performed on the same treadmill. The speed was set to 9 km/h for the first 3 min and was then increased by 0.5 km/h every 30 s. Then, after a short break (< 5 min), three 1-min runs (9, 11, 13 km/h) were performed in a randomized order (1-min recovery between each run where runners just stand). 3D kinematic and kinetic data were collected during the static trial and the first 10 strides following the 30-s mark of the running trials. All participants were familiar with running on a treadmill, as it was part of their usual training program, and they wore their habitual running shoes during testing (shoe mass: 257 ± 49 g and shoe heel-to-toe drop: 7 ± 3 mm).

### Data collection

Whole-body 3D kinematic data were collected at 200 Hz using motion capture (8 cameras) and Vicon Nexus software v2.9.3 (Vicon, Oxford, UK). The laboratory coordinate system was oriented such that the *x*-, *y*-, and *z*-axes denoted the mediolateral (pointing towards the right side of the body), posterior-anterior, and inferior-superior axes, respectively. Forty-three and 39 retroreflective markers of 12.5 mm diameter were used for the static and running trials, respectively. They were affixed to the skin and shoes of the individuals on anatomical landmarks using double-sided tape following standard guidelines^26^. Synchronized kinetic data (1000 Hz) were also collected using the force plate embedded into the treadmill.

The 3D marker and ground reaction force data (analog signal) were exported in the .c3d format and processed in Visual3D Professional software v6.01.12 (C-Motion Inc., Germantown, MD, USA). The 3D marker data were interpolated using a third-order polynomial least-square fit algorithm (using three frames of data before and after the “gap” to calculate the coefficients of the polynomial), allowing a maximum of 20 frames for gap filling, and were subsequently low-pass filtered at 20 Hz using a fourth-order Butterworth filter. The 3D ground reaction force signal was filtered using the same filter^27^ and down sampled to 200 Hz to match the sampling frequency of the marker data.

### Data analysis

For each running trial, the foot-strike (FS) and toe-off (TO) events were identified with Visual3D. These events were detected by applying a 20 N threshold to the *z-*component of the ground reaction force^28^. More explicitly, FS was detected as the first data point greater than or equal to 20 N within a running step, while TO was detected at the last data point greater than or equal to 20 N within the same running step. ${t}_{c}$ and ${t}_{s}$ were defined as the times from FS to TO and from TO to FS of the same foot, respectively. DF was calculated as follows^21^:

1 $${\text{DF}} = \frac{{t_{c} }}{{t_{c} + t_{s} }} = t_{c} {\text{ SF}},$$

where SF denotes the stride frequency. In addition, a full-body biomechanical model with six degrees of freedom and 15 rigid segments was constructed from the marker set. The segments included the head, upper arms, lower arms, hands, thorax, pelvis, thighs, shanks, and feet. In Visual3D, the segments were treated as geometric objects, assigned inertial properties and center of mass locations based on their shape^29^, and attributed relative masses based on standard regression equations^30^. The foot segment angle was defined as the angle of the foot segment relative to the laboratory coordinate system and computed using an *x–y–z* Cardan sequence. The foot segment was obtained using five markers which were placed at the apex of both the lateral and medial malleolus, foot calcaneus (aspect of the Achilles tendon insertion), and head of the first and fifth metatarsals. The *x*-component of the foot segment angle at FS was used to determine FSP following the procedure proposed by Altman and Davis^2^. In brief, the average foot segment angle of the standing static trial was subtracted from that of running trials such that 0° corresponded to a foot parallel to the ground. Then, the angle at FS, i.e., FSA, was computed using the *x*-component of the rescaled foot segment angle (negative and positive angle values represented plantar flexion and dorsiflexion, respectively).

For all biomechanical measures, the values extracted from the 10 strides for each participant were averaged for subsequent analyses. Data analysis was performed using Python (v3.7.4, available at http://www.python.org).

### Runners’ classification

High (DF_high_), mid (DF_mid_), and low (DF_low_) DF groups were created using the terciles of the group (i.e., the 33 highest, 33 middle, and 34 lowest DF values at each speed). Of note, DF_low_ group was composed of one extra runner but attributing this extra runner to DF_mid_ or DF_high_ group or removing their data from the study would not have had an impact on the results. In addition, runners were classified as RFS, MFS, and FFS if FSA values were ≥ 8°, < 8° but ≥  − 1.6°, and <  − 1.6°, respectively, at each speed^2^. A similar analysis was also performed using FSP groups created based on an absolute classification of runners, i.e., RFS, MFS, and FFS being represented by the 33 highest, 33 middle, and 34 lowest FSA values at each speed, and is presented in section S1 of supplementary materials. The relative^2^ and absolute classifications to create FSP groups led to similar results because both classifications classified most of the runners in the same group. Indeed, on average, 1 participant (4%) was attributed to a different FSP group when using the absolute rather than the relative classification.

### Statistical analysis

All data are presented as the mean ± standard deviation. A chi-squared test was used to compare the foot-strike distribution at the different speeds.

Then, after the residual plots were inspected, and no obvious deviations from homoscedasticity or normality were observed, a linear mixed model fitted by restricted maximum likelihood was used to compare DF values for the different FSP groups and speeds. The within-subject nature was controlled for by including random effects for participants. Pairwise post hoc comparisons were performed using Holm corrections. The differences between groups were quantified using Cohen’s *d* effect size^31^. The effect sizes were interpreted as very small, small, moderate, or large when |*d*| values were close to 0.01, 0.2, 0.5, or 0.8, respectively^31^.

A similar linear mixed model was used to compare FSA values for the different DF groups and speeds. Linear mixed models were also used to compare DF and FSA values among DF and FSP groups (considering all groups together) and speeds. These tests were used to investigate the difference in DF and FSA values between the three group pairs (RFS and DF_high_, MFS and DF_mid_, FFS and DF_low_). Therefore, only the group x running speed interaction effect was investigated, and, if significant, the pairwise comparisons between these three group pairs at each running speed were reported.

Agreement between FSP and DF groups as well as sensitivity and specificity of the agreement were calculated for the three speeds^32^. As participants were classified in three FSP and DF groups, agreement, sensitivity, and specificity were obtained for each of the three group pairs by collapsing to three 2 × 2 classifications, i.e., RFS and DF_high_ vs non-RFS and non-DF_high_, MFS and DF_mid_ vs non-MFS and non-DF_mid_, and FFS and DF_low_ vs non-FFS and non-DF_low_. Agreement was defined as the sum of the number of runners in a DF group that were attributed to the corresponding FSP group and the number of runners in the corresponding non-DF group that were attributed to the non-FSP group over the total number of runners, e.g., the sum of DF_high_ runners in RFS and non-DF_high_ runners in non-RFS over all runners. Sensitivity was defined as the number of runners in a DF group that were attributed to the corresponding FSP group over the total number of runners in the corresponding FSP group, e.g., DF_high_ runners among RFS. Specificity was defined as the number of runners in a non-DF group that were attributed to the corresponding non-FSP group over the total number of runners in the corresponding non-FSP group, e.g., non-DF_high_ runners among non-RFS. The 95% confidence intervals (lower, upper) of the agreement between FSP and DF groups and of the sensitivity and specificity values, were estimated using binomial exact calculation.

The Pearson’s correlation coefficient (*r*) and its corresponding 95% confidence interval (lower, upper) and *P*-values were computed for the relation between FSA and DF, as well as ${t}_{c}$ and SR, i.e., the variables constituting DF, for the three speeds. In addition, correlations among shoe mass, shoe heel-to-toe drop, DF, and FSA were computed to investigate if footwear could affect DF and FSA. Very high, high, moderate, low, and negligible correlations were given by |*r|* values of 0.90–1.00, 0.70–0.90, 0.50–0.70, 0.30–0.50, and 0.00–0.30, respectively^33^.

Statistical analysis was performed using Jamovi (v1.2, retrieved from https://www.jamovi.org) with a level of significance set at *P* ≤ 0.05.

## Results

### Distribution of runners within foot-strike pattern groups

The number of RFS, MFS, and FFS together with their corresponding FSAs at all speeds examined are given in Table 1. The chi-squared test showed no differences in the foot-strike distribution at the different speeds employed (${\chi }^{2}=4.6$, *P* = 0.34), revealing homogeneity among groups at all speeds. On average, 2 participants per group (7%) changed their FSP group with running speed while 4 participants per group (12%) changed their DF group. The complete analysis is provided in section S2 of supplementary materials.

**Table 1:** Number of rearfoot (RFS), midfoot (MFS), and forefoot (FFS) strikers observed in the cohort of participants (*N* = 100) and their corresponding foot-strike angles at three running speeds.

	RFS		MFS		FFS	
Running speed (km/h)	Count	Angle (°)	Count	Angle (°)	Count	Angle (°)
9	27	13.3 ± 2.9	34	4.3 ± 4.2	39	− 6.7 ± 4.7
11	31	13.2 ± 2.8	33	3.4 ± 2.7	36	− 7.7 ± 3.3
13	38	11.7 ± 4.1	23	1.4 ± 4.1	39	− 5.9 ± 5.6

The values are presented as the mean ± standard deviation.

### Duty factor values within foot-strike pattern groups

The linear mixed model revealed a significant FSP group effect on DF (*P* < 0.001). The Holm post hoc tests indicated a significantly higher DF for RFS than for MFS and FFS (*P* ≤ 0.005), and for MFS than for FFS (*P* = 0.001). A significant effect of speed was reported on DF (*P* < 0.001). A significantly smaller DF was obtained at a faster speed, as depicted by the Holm post hoc tests (*P* < 0.001). There was no FSP group x speed interaction (*P* < 0.66). The Cohen’s *d* effect sizes were moderate (|*d*|≤ 0.66), except for those corresponding to the RFS-FFS pairs, which were large at all speeds (|*d*|≥ 0.96).

### Foot-strike angle values within duty factor groups

The DF ranges for DF_low_, DF_mid_, and DF_high_ groups were [31.6%, 36.3%], [36.4%, 38.4%], and [38.6%, 45.3%] at 9 km/h, [28.5%, 33.4%], [33.4%, 35.7%], and [35.8%, 40.2%] at 11 km/h, and [27.0%, 31.4%], [31.5%, 33.5%], and [33.5%, 37.6%] at 13 km/h, respectively. The linear mixed model revealed a significant DF group effect on FSA (*P* < 0.001). The Holm post hoc tests indicated a significantly higher FSA for DF_high_ than for DF_mid_ and DF_low_ (*P* < 0.001), and for DF_mid_ than for DF_low_ (*P* = 0.005). A significant effect of speed was reported on FSA (*P* < 0.001). A significantly higher FSA was obtained at a faster speed, as reported by the Holm post hoc tests (*P* ≤ 0.01). There was no DF group x speed interaction (*P* < 0.42). The Cohen’s *d* effect sizes were moderate (|*d*| ≤ 0.68), except for those corresponding to the DF_high_-DF_low_ pairs, which were large at all speeds (|*d*| ≥ 0.86).

### Duty factor and foot-strike angle values within all (duty factor and foot-strike pattern) groups together

When considering all groups together, a significant group x running speed interaction effect was reported by the linear mixed models for both DF and FSA values (*P* ≤ 0.013). Pairwise post hoc comparisons between the three group pairs (RFS and DF_high_, MFS and DF_mid_, FFS and DF_low_) at each running speed revealed no significant differences for DF and FSA values (*P* ≥ 0.16).

### Agreement between foot-strike pattern and duty factor groups

The number of runners in FSP and DF groups as well as the agreement, sensitivity, and specificity between FSP and DF groups are given in Table 2. The average (over speed and group) agreement, sensitivity, and specificity were 73, 49, and 75%, respectively.

**Table 2:** Number of runners in foot-strike pattern (FSP) [rearfoot (RFS), midfoot (MFS), and forefoot (FFS) strikers] and duty factor (DF) [high (DF_high_), mid (DF_mid_), and low (DF_low_) DF runners] groups, as well as the agreement, sensitivity, and specificity between FSP and DF groups together with their 95% confidence intervals in parentheses (lower, upper) at three running speeds.

Running speed (km/h)		DF_high_	DF_mid_	DF_low_
9	RFS	15	8	4
	MFS	11	12	11
	FFS	7	13	19
	Agreement (%)	70 (61, 79)	76 (68, 84)	65 (56, 74)
	Sensitivity (%)	56 (37, 74)	35 (19, 51)	49 (33, 64)
	Specificity (%)	75 (65, 85)	68 (57, 79)	75 (65, 86)
11	RFS	18	8	5
	MFS	9	14	10
	FFS	6	11	19
	Agreement (%)	72 (63, 81)	81 (73, 89)	68 (59, 77)
	Sensitivity (%)	58 (41, 75)	42 (26, 59)	53 (36, 69)
	Specificity (%)	78 (69, 88)	72 (61, 82)	77 (66, 87)
13	RFS	21	9	8
	MFS	5	11	7
	FFS	7	13	19
	Agreement (%)	71 (62, 80)	85 (78, 92)	65 (56, 74)
	Sensitivity (%)	55 (39, 71)	48 (27, 68)	49 (33, 64)
	Specificity (%)	81 (71, 90)	71 (61, 82)	75 (65, 86)

The DF and FSA values of runners attributed to a DF group but not being classified in the supposedly corresponding FSP group, for instance DF_high_ runners but classified as MFS and FFS, are given in Fig. 2A. Similarly, Fig. 2B depicts FSA and DF values of runners attributed to a FSP group but not being classified in the supposedly corresponding DF group, for instance RFS but classified as DF_mid_ or DF_low_.

### Relationships between foot-strike angle and duty factor, contact time, and stride frequency

The correlations between FSA and DF, ${t}_{c}$, and SF, together with their 95% confidence intervals, are given in Table 3. For DF and ${t}_{c}$, the correlation was weak (low) but statistically significant (*r* ≤ 0.50; *P* < 0.001) for all speeds, while the correlation between DF and SF was negligible and not statistically significant (*|r*|≤ 0.14; *P* ≥ 0.18).

**Table 3:** Pearson’s correlation coefficients (*r*) and the corresponding 95% confidence intervals (lower, upper) and *P*-values for the relationships between the foot-strike angle and duty factor (DF), contact time (${t}_{c}$), and stride frequency (SF) for three tested speeds.

	Running Speed (km/h)	r	*P*
DF	9	0.39 (0.21, 0.55)	** < 0.001**
	11	0.42 (0.24, 0.57)	** < 0.001**
	13	0.48 (0.31, 0.62)	** < 0.001**
*t*_c_	9	0.43 (0.26, 0.58)	** < 0.001**
	11	0.47 (0.30, 0.61)	** < 0.001**
	13	0.50 (0.34, 0.63)	** < 0.001**
SF	9	− 0.13 (− 0.32, 0.06)	0.18
	11	− 0.14 (− 0.28, 0.11)	0.36
	13	− 0.11 (− 0.30, 0.09)	0.29

The statistically significant correlations (*P* ≤ 0.05) are indicated in bold font.

### Relationships between shoe mass, shoe heel-to-toe drop, foot-strike angle, and duty factor

The correlation between shoe mass and shoe heel-to-toe drop was low but significant [*r* = 0.52 (0.37, 0.65); *P* < 0.001]. However, the correlations between shoe mass and DF, shoe heel-to-toe drop and DF, shoe mass and FSA, and shoe heel-to-toe drop and FSA were negligible and not statistically significant (|*r*| ≤ 0.18; *P* ≥ 0.08; Table 4) except between DF and shoe mass at 13 km/h (*r* = 0.20; *P* = 0.04) and between FSA and shoe heel-to-toe drop at 9 km/h (*r* = 0.21; *P* = 0.04) which were significant.

**Table 4:** Pearson’s correlation coefficients (*r*) and the corresponding 95% confidence intervals (lower, upper) and *P*-values for the relationships among shoe mass, shoe heel-to-toe drop, foot-strike angle (FSA), and duty factor (DF) for three tested speeds.

Variables	Running speed (km/h)	Shoe mass		Shoe heel-to-toe drop	
		r	*P*	R	*P*
DF	9	0.13 (− 0.07, 0.32)	0.21	0.14 (− 0.06, 0.33)	0.16
	11	0.13 (− 0.07, 0.32)	0.2	0.14 (− 0.06, 0.33)	0.16
	13	0.20 (0.01, 0.38)	**0.04**	0.11 (− 0.09, 0.30)	0.26
FSA	9	0.18 (− 0.02, 0.36)	0.08	0.21 (0.01, 0.39)	**0.04**
	11	0.15 (− 0.05, 0.34)	0.14	0.16 (− 0.03, 0.35)	0.1
	13	0.18 (− 0.02, 0.36)	0.08	0.16 (− 0.04, 0.34)	0.12

The statistically significant correlations (*P* ≤ 0.05) are indicated in bold font.

## Discussion

The purpose of the present study was to compare two different classification methods (either based on DF or FSA) in analyzing running gait at several running speeds. In the present study, a significantly higher DF was obtained for RFS than for MFS and FFS and for MFS than for FFS, supporting our first hypothesis. Moreover, a significantly higher FSA was reported for DF_high_ than for DF_mid_ and DF_low_ and for DF_mid_ than for DF_low_, supporting our second hypothesis. Furthermore, the three group pairs (RFS and DF_high_, MFS and DF_mid_, FFS and DF_low_) did not report any significant difference in DF and FSA values at each tested speed. However, although weak correlations were obtained between FSA and DF values, the agreement between FSP and DF groups was 73%, which did not fully support our third hypotheses. Nonetheless, the sensitivity between FSP and DF groups was 50%, meaning that only one in two runners was attributed to the DF group supposedly corresponding to the FSP group. Therefore, although DF and FSA values were not statistically different between each of the three group pairs (at a group level), the runners constituting these groups were not the same in 50% of the cases and DF should be preferred to FSP/FSA when evaluating the global running pattern of a runner.

### Homogeneous foot-strike pattern distribution

The groups created based on FSP were homogeneous at all speeds, although FSP was not a criterion for recruiting participants. Larson, et al.^34^ reported that ~ 90% of recreational runners in a road race were RFS, which makes the FSP distribution of ~ 33% observed for each group surprising and unexpected (Table 1). One possible explanation is that the participants of this study followed the popular advice given by coaches over the past decade promoting a more mid- to forefoot pattern than a rearfoot strike pattern^35–37^. Although recent reviews^15,18^ concluded that there is no scientific foundation to recommend non-injured rearfoot strikers to change their RFS. Another explanation is the young age of the participants of this study (30 ± 7 years). In fact, older people were shown to run with a more rearfoot strike pattern than younger people^38,39^. Finally, though shoe mass and shoe heel-to-toe drop were not associated to DF and FSA (Table 4), other footwear characteristics not assessed as part of this study could impact DF or FSA values, such as midsole cushioning and/or the longitudinal bending stiffness^40^. Nevertheless, the homogeneity of the FSP groups made the results of this study more robust when comparing FSP groups due to similar group sizes.

### Duty factor and foot-strike pattern differ at the individual level

**Figure 1:**
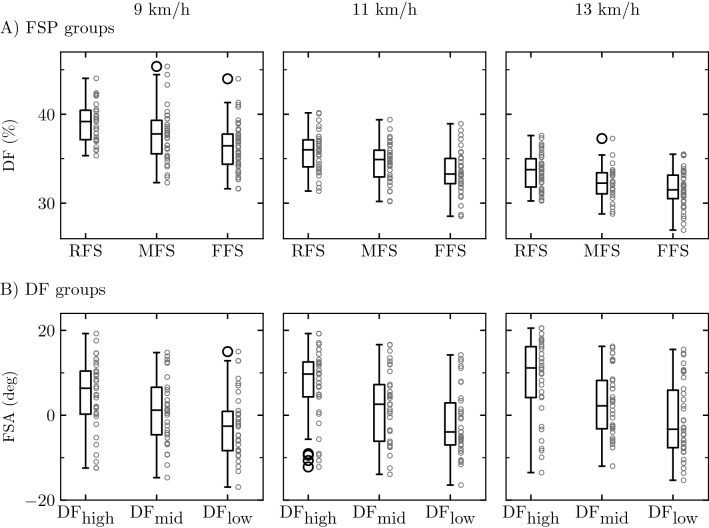
Boxplots of (**A**) the duty factor (DF) for the different foot-strike pattern (FSP) groups, i.e., rearfoot (RFS), midfoot (MFS), and forefoot (FFS) strikers, and (**B**) the foot-strike angle (FSA) for the different DF groups, i.e., high (DF_high_), mid (DF_mid_), and low (DF_low_) DF runners, at 9, 11, and 13 km/h. The box extends from the lower to upper quartile values of the data, with a line at the median. The whiskers extend from the box to show the range of the data while flier points (black empty circles) are those past the end of the whiskers. The upper whisker extends to the last data less than Q3 + 1.5 (Q3 – Q1), where Q1 and Q3 are the first and third quartile. Similarly, the lower whisker extends to the first data greater than Q1 – 1.5 (Q3 – Q1). The small gray empty circles denote the data of each participant.

DF was significantly lower for FFS than for RFS and MFS and for MFS than for RFS, with a moderate to large effect size (Fig. 1). These results confirm previous observations that there should be a trend towards a more forefoot strike pattern with a decreasing DF value^19,20^. Similarly, FSA was significantly lower for DF_low_ than for DF_high_ and DF_mid_ and for DF_mid_ than for DF_high_, with also a moderate to large effect size (Fig. 1). Moreover, no significant difference was revealed between the three group pairs at each tested speed. However, the sensitivity between DF and FSP groups was 50%, reflecting that only one in two runners in a DF group (50%) were classified in the supposedly corresponding FSP group, although there was a slightly greater chance of matching among RFS (56%) and FFS (50%) than among MFS (42%).

**Figure 2:**
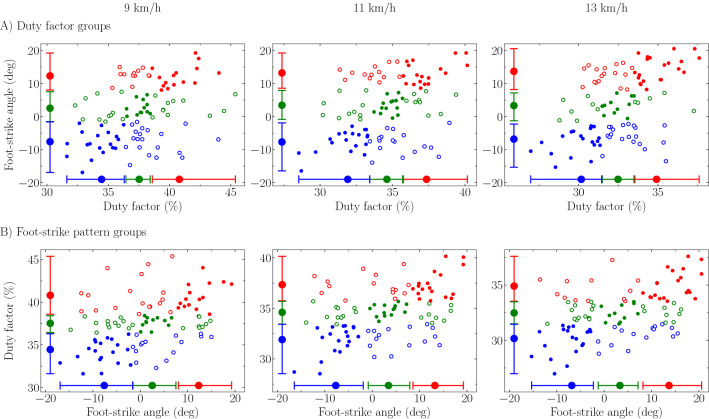
Duty factor (DF) and foot-strike angle (FSA) values of runners attributed to (**A**) a DF group but not being classified in the supposedly corresponding foot-strike pattern (FSP) group and (**B**) a FSP group but not being classified in the supposedly corresponding DF group at each tested running speed. Mean DF and FSA value (filled circle) and range of values (whiskers) for each DF and FSP group, i.e., high DF runners and rearfoot strikers (RFS; red), mid DF runners and midfoot strikers (MFS; green), and low DF runners and forefoot strikers (FFS; blue). The upper whisker extends to the maximum while the lower whisker extends to the minimum value. Empty circles denote the runners attributed to a DF or FSP group but not being classified in the supposedly corresponding FSP or DF group, respectively, e.g., high DF runners but classified as MFS or FFS (green and blue empty circles within the red whiskers of the high DF runners) in (**A**) and RFS but classified as mid or low DF runners (green and blue empty circles within the red whiskers of RFS) in (**B**).

This might be explained by the fact that the DF range corresponding to DF_mid_ runners and FSA range corresponding to MFS are smaller than the DF ranges corresponding to DF_high_ and DF_low_ runners and FSA ranges corresponding to RFS and FFS. Besides, the DF values of runners attributed to a DF group but not being classified in the supposedly corresponding FSP group mostly span the entire range of DF values of this DF group (Fig. 2A). A similar observation is made for FSA values of runners attributed to a FSP group but not being classified in the supposedly corresponding DF group (Fig. 2B). Thereby, these results suggest that ‘local’ FSP/FSA and DF do not represent similar running pattern information when investigated at the individual level.

### Weak association between duty factor and foot-strike angle

Weak but significant correlations were observed between DF and FSA at all speeds (*r* ≤ 0.48 and *P* < 0.001; Table 3). Nonetheless, FSA was only able to explain ~ 20% of the variance of DF. The angle of the lower limb at initial ground contact relative to the vertical axis^41^ can be estimated using ${t}_{c}$ and therefore DF (indirectly). In addition, according to the observations of Breine et al.^42^ which showed that RFS have a less vertical leg at the point of contact than do runners landing further forward on their foot (MFS and FFS), FSP is indirectly related to the lower limb angle at initial contact. As RFS position their foot to be much more forward than their pelvis to strike the ground with their heel, these runners have a higher lower limb angle at initial contact than do FFS. Therefore, the lower limb angle at initial contact may be indirectly related to FSA. Hence, there is an indirect relationship between FSA and DF which is supported by the indirect relationship between the lower limb angle at initial contact and both DF and FSA. Besides, the 50% sensitivity reported between FSP and DF groups can be partly explained by the weak correlations between DF and FSA, which also corroborate that FSP represents only a portion of DF. Indeed, DF is computed from ${t}_{c}$ and SF (Eq. 1), which makes it to be functionally representative of a more global biomechanical behavior^19,20,24^. For instance, DF has been shown to represent the trade-off between muscle contractile mechanics and energetics in running as a valid estimate of the muscle force–length-velocity related to mechanical work, total active muscle volume, and energy expenditure in running^24^.

Correlation coefficients between DF and FSA increased with increasing running speed (+ 20% from 9 to 13 km/h; Table 3), depicting that FSA was more strongly correlated with DF with increasing speed. These results suggest that FSA and DF should be more similar at faster speeds. This might partly be attributed to the smaller ranges of DF and FSA values with increasing speed. Nonetheless, the present study did not report an increase in sensitivity with increasing speed except for DF_mid_ runners (Table 2). The increase in sensitivity for DF_mid_ runners could partly be explained by the fact the DF range of DF_mid_ runners relatively increased compared to the DF ranges of DF_high_ and DF_low_ runners with increasing speed. Nevertheless, the relation between FSP and DF groups as well as FSA and DF values at faster running speeds should further be investigated.

The correlations between ${t}_{c}$ and FSA were weak but statistically significant and slightly stronger than those between DF and FSA (+ 4%; Table 3). Nonetheless, FSA was only able to explain up to 25% of the variance of ${t}_{c}$, confirming that ${t}_{c}$ (as DF) does not only represent what happens at initial contact with the ground as does FSP. The weaker correlation between DF and FSA than that between ${t}_{c}$ and FSA can be explained by the negligible correlations between SF and FSA (*|r*|≤ 0.14; Table 3) coupled to the fact that DF is given by the product between ${t}_{c}$ and SF (Eq. 1).

### Limitations

A few limitations of the present study exist. An unexpected high proportion of runners were classified as FFS, indicating that the study population may not be representative of the general population. The speeds were limited to endurance speeds, and running trials were only performed on a treadmill. Furthermore, participants wore their own running shoes during testing, which could be confounding our results. Given that differences in footwear characteristics can underpin differences in running biomechanics^43^, using a standardized shoe might have led to different study outcomes in terms of FSA and DF. Noteworthy, however, is that there were no significant correlations between shoe mass and DF and FSA and between shoe heel-to-toe drop and DF and FSA. Recreational runners are more comfortable wearing their own shoes^44^, and show individual responses to novel footwear^44,45^ and cushioning properties^46^. Nevertheless, it is possible that other footwear characteristics not assessed as part of this study correlate to DF or FSA, such as midsole cushioning and/or the longitudinal bending stiffness^40^. Moreover, very few studies on DF exist. Therefore, it is difficult to determine how DF may be affected by confounding variables such as footwear or the running surface. Therefore, future studies should focus on the relation between DF and FSP under additional conditions (i.e., faster speeds, different types of ground, and different shoes). Nonetheless, the presented results are strong due to the use of a large dataset.

## Conclusion

This study revealed that RFS depict higher DF than MFS and FFS and similarly for MFS than FFS. Moreover, DF_high_ showed higher FSA than DF_mid_ and DF_low_ and similarly for DF_mid_ than DF_low_. However, weak correlations were obtained between FSA and DF values as well as a sensitivity of 50% between FSP and DF groups, meaning that only one in two runners was attributed to the DF group supposedly corresponding to the FSP group. Therefore, though DF and FSA values were not statistically different between each of the three group pairs (at a group level), these results suggest that the runners constituting these groups were not the same. In other words, ‘local’ FSP/FSA and DF do not represent similar running pattern information when investigated at an individual level and DF should be preferred to FSP/FSA when evaluating the global running pattern of a runner.

## Supplementary Information

Supplementary Information.

## Data Availability

The datasets supporting this article are available upon request by the corresponding author.
