# Supplementary material for: Duty factor and foot-strike pattern do not represent similar running pattern at the individual level
Source: Sci Rep. 2022 Jul 29;12:13061. doi: 10.1038/s41598-022-17274-0 (PMC9338241; doi:10.1038/s41598-022-17274-0)
Supplement: Supplementary file 1 — Supplementary Information. [file 41598_2022_17274_MOESM1_ESM.docx]

*Supplementary Materials for:*

**Duty factor and foot-strike pattern do not represent similar running pattern at the individual level**

**S1. Absolute classification of runners to create foot-strike pattern groups**

Runners were classified as rearfoot (RFS), midfoot (MFS), and forefoot (FFS) strikers using the 33 highest, 33 middle, and 34 lowest foot-strike angle (FSA) values at each speed. Noteworthy, FFS group was composed of one extra runner but attributing this extra runner to MFS or RFS group or removing him from the study would not have had an impact on the results.

Both relative and absolute classifications classified most of the runners in the same foot-strike pattern (FSP) group. On average, 1 participant (4%) was attributed to a different FSP group when using the absolute rather than the relative classification reported in the manuscript. The complete analysis of the number of runners that were attributed to a different FSP group between the relative and absolute classifications at the different running speeds is provided in Table S1.

**Table S1.** Number and percentage (in parentheses) of runners that were attributed to a different foot-strike pattern (FSP) group, i.e., rearfoot (RFS), midfoot (MFS), or forefoot (FFS) group, between the relative and absolute classifications at the different running speeds.

| **Relative classification** | **Absolute classification** | **9 km/h** | **11 km/h** | **13 km/h** |
| --- | --- | --- | --- | --- |
| FFS | MFS | 5 (15%) | 2 (6%) | 5 (15%) |
| MFS | FFS | 0 (0%) | 0 (0%) | 0 (0%) |
| MFS | RFS | 6 (18%) | 2 (6%) | 0 (0%) |
| RFS | MFS | 0 (0%) | 0 (0%) | 5 (15%) |
| FFS | RFS | 0 (0%) | 0 (0%) | 0 (0%) |
| RFS | FFS | 0 (0%) | 0 (0%) | 0 (0%) |

The FSA ranges for FFS, MFS, and RFS groups were [-16.9°, -2.7°], [-2.5°, 5.5°], and [6.0°, 19.3°] at 9km/h, [-16.9°, -3.5°], [-2.9°, 7.7°], and [7.8°, 19.2°] at 11km/h, and [-15.3°, -3.0°], [-2.9°, 9.7°], and [9.9°, 20.5°] at 13km/h, respectively. The linear mixed model revealed a significant FSP group effect on duty factor (DF) (*P* < 0.001). The Holm post hoc tests indicated a significantly higher DF for RFS than for MFS and FFS (*P* ≤ 0.001), and for MFS than for FFS (*P* = 0.004). A significant effect of speed was reported on DF (*P* < 0.001). A significantly smaller DF was obtained at a faster speed, as depicted by the Holm post hoc tests (*P* < 0.001). There was no FSP group x speed interaction (*P* < 0.81). The Cohen’s *d* effect sizes were moderate (|*d*| ≤ 0.62), except for those corresponding to the RFS-FFS pairs, which were large at all speeds (|*d*| ≥ 0.92).

When considering all groups together, a significant group x running speed interaction effect was reported by the linear mixed models for both DF and FSA values (*P* ≤ 0.018). Pairwise post hoc comparisons between the three group pairs at each running speed revealed no significant differences for DF and FSA values (P ≥ 0.18).

The number of runners in FSP and DF groups as well as the agreement, sensitivity, and specificity between FSP and DF groups are given in Table S2. The average (over speed and group) agreement, sensitivity, and specificity were 72, 50, and 75%, respectively.

**Figure S1.** Boxplots of the duty factor (DF) for the different foot-strike pattern groups, i.e., rearfoot (RFS), midfoot (MFS), and forefoot (FFS) strikers, at 9, 11, and 13 km/h. The box extends from the lower to upper quartile values of the data, with a line at the median. The whiskers (black empty circles) extend from the box to show the range of the data while flier points are those past the end of the whiskers. The upper whisker extends to the last data less than Q3 + 1.5 (Q3 – Q1), where Q1 and Q3 are the first and third quartile. Similarly, the lower whisker extends to the first data greater than Q1 – 1.5 (Q3 – Q1). The small gray empty circles denote the data of each participant.

The DF and FSA values of runners attributed to a DF group but not being classified in the supposedly corresponding FSP group, for instance DF_high_ runners but classified as MFS and FFS, are given in Fig. S2A. Similarly, Fig. S2B depicts FSA and DF values of runners attributed to a FSP group but not being classified in the supposedly corresponding DF group, for instance RFS but classified as DF_mid_ or DF_low_.

On average, 2 participants (6%) changed their FSP group with running speed. The complete analysis of the number of runners that switched group between the different running speeds is provided in Table S3.

**Table S2.** Number of runners in foot-strike pattern (FSP) [rearfoot (RFS), midfoot (MFS), and forefoot (FFS) strikers] and duty factor (DF) [high (DF_high_), mid (DF_mid_), and low (DF_low_) DF runners] groups, as well as the agreement, sensitivity, and specificity between FSP and DF groups together with their 95% confidence intervals in parenthesis at three running speeds.

| **Running Speed (km/h)** |  | **DF_high_** | **DF_mid_** | **DF_low_** |
| --- | --- | --- | --- | --- |
| 9 | RFS | 17 | 10 | 6 |
|  | MFS | 10 | 12 | 11 |
|  | FFS  Agreement (%)  Sensitivity (%)  Specificity (%) | 6  68 (59, 77)  52 (34, 69)  76 (66, 86) | 11  75 (67, 83)  36 (20, 53)  69 (58, 80) | 17  66 (57, 75)  50 (33, 67)  74 (64, 85) |
| 11 | RFS | 18 | 8 | 77 |
|  | MFS | 10 | 14 | 9 |
|  | FFS  Agreement (%)  Sensitivity (%)  Specificity (%) | 5  70 (61, 79)  55 (38, 72)  78 (68, 88) | 11  80 (72, 88)  42 (26, 59)  72 (61, 82) | 18  68 (59, 77)  53 (36, 70)  76 (65, 86) |
| 13 | RFS | 19 | 7 | 7 |
|  | MFS | 8 | 16 | 9 |
|  | FFS  Agreement (%)  Sensitivity (%)  Specificity (%) | 6  72 (63, 81)  58 (41, 74)  79 (69, 89) | 10  84 (77, 91)  48 (31, 66)  75 (64, 85) | 18  68 (59, 77)  53 (36, 70)  76 (65, 86) |

**Table S3.** Number and percentage (in parentheses) of runners that changed from one foot-strike pattern (FSP) group, i.e., rearfoot (RFS), midfoot (MFS), or forefoot (FFS) group, to another FSP group with changing running speed.

|  | **9 to 11 km/h** | **11 to 13 km/h** | **9 to 13 km/h** |
| --- | --- | --- | --- |
| **FFS to MFS** | 1 (3%) | 5 (15%) | 3 (9%) |
| **MFS to FFS** | 3 (9%) | 3 (9%) | 3 (9%) |
| **MFS to RFS** | 2 (6%) | 3 (9%) | 2 (6%) |
| **RFS to MFS** | 4 (12%) | 1 (3%) | 2 (6%) |
| **FFS to RFS** | 1 (3%) | 0 (0%) | 0 (0%) |
| **RFS to FFS** | 0 (0%) | 2 (6%) | 1 (3%) |

**Figure S2.** Duty factor (DF) and foot-strike angle (FSA) values of runners attributed to (A) a DF group but not being classified in the supposedly corresponding foot-strike pattern (FSP) group and (B) a FSP group but not being classified in the supposedly corresponding DF group at each tested running speed. Mean DF and FSA value (filled circle) and range of values (whiskers) for each DF and FSP group, i.e., high DF runners and rearfoot strikers (RFS; red), mid DF runners and midfoot strikers (MFS; green), and low DF runners and forefoot strikers (FFS; blue). The upper whisker extends to the maximum while the lower whisker extends to the minimum value. Empty circles denote the runners attributed to a DF or FSP group but not being classified in the supposedly corresponding FSP or DF group, respectively, e.g., high DF runners but classified as MFS or FFS (green and blue empty circles within the red whiskers of the high DF runners) in (A) and RFS but classified as mid or low DF runners (green and blue empty circles within the red whiskers of RFS) in (B).

**S2. Runners attributed to a different foot-strike pattern and duty factor group depending on the running speed**

**Table S4.** Number and percentage (in parentheses) of runners that changed from one foot-strike pattern (FSP) group, i.e., rearfoot (RFS), midfoot (MFS), or forefoot (FFS) group, to another FSP group with changing running speed.

|  | **9 to 11 km/h** | **11 to 13 km/h** | **9 to 13 km/h** |
| --- | --- | --- | --- |
| **FFS to MFS** | 4 (10%) | 2 (5%) | 3 (8%) |
| **MFS to FFS** | 1 (3%) | 4 (12%) | 2 (6%) |
| **MFS to RFS** | 5 (15%) | 8 (24%) | 12 (35%) |
| **RFS to MFS** | 1 (4%) | 0 (0%) | 0 (0%) |
| **FFS to RFS** | 0 (0%) | 0 (0%) | 0 (0%) |
| **RFS to FFS** | 0 (0%) | 1 (4%) | 1 (4%) |

**Table S5.** Number and percentage (in parentheses) of runners that changed from one duty factor (DF) group, i.e., high (DF_high_), mid (DF_mid_), or low (DF_low_) DF, to another DF group with changing running speed.

|  | **9 to 11 km/h** | **11 to 13 km/h** | **9 to 13 km/h** |
| --- | --- | --- | --- |
| **DF_low_ to DF_mid_** | 6 (18%) | 6 (18%) | 6 (18%) |
| **DF_mid_ to DF_low_** | 6 (18%) | 5 (15%) | 6 (18%) |
| **DF_mid_ to DF_high_** | 5 (15%) | 5 (15%) | 7 (21%) |
| **DF_high_ to DF_mid_** | 5 (15%) | 4 (12%) | 7 (21%) |
| **DF_low_ to DF_high_** | 0 (0%) | 0 (0%) | 1 (3%) |
| **DF_high_ to DF_low_** | 0 (0%) | 1 (3%) | 1 (3%) |
